# Pro-Environmental Behaviour: Psychometric assessment and relationship with psychological variables in a Portuguese community sample

**DOI:** 10.1192/j.eurpsy.2025.1807

**Published:** 2025-08-26

**Authors:** C. Cabaços, A. Macedo, M. J. Soares, A. I. Araújo, A. S. Grave, A. T. Pereira

**Affiliations:** 1 Institute of Psychological Medicine, Faculty of Medicine; 2 Coimbra Institute for Biomedical Imaging and Translational Research, University of Coimbra, Coimbra, Portugal

## Abstract

**Introduction:**

Climate change is one of the main global challenges of the 21^st^ century due to its consequences for the environment, the economy and health. Given that human behaviour exists at the forefront of many of the environmental issues we face, it is crucial to effectively measure pro-environmental behaviours (PEB). Stanley et al. (2021) proposed the PEB Scale (PEBS), a bifactorial measure including eight items asking about personal behaviours and eight collective actions that revealed good psychometric properties. To the best of our knowledge, there is no instrument that validly assesses these parameters in the general Portuguese population.

**Objectives:**

To analyze the psychometric properties of the Portuguese version of PEBS and to explore its relationship with individual factors.

**Methods:**

A community sample of 599 Portuguese adults (64.6% women; mean age=34.40±16.18) answered to the Portuguese preliminary version of PEBS and to the validated instruments: Climate Change Distress and Impairment Scale/CC-DIS, Big3 Perfectionism Scale–Short version/BTPS-SF, HEXACO-60 and Toronto and Coimbra Prosocial Behaviour Questionnaire/ProBeQ. SPSS 29 and AMOS-29 were used for Exploratory Factor Analysis (EFA; with a subsample of n=291) and Confirmatory Factor Analysis (CFA; n=308), respectively.

**Results:**

EFA revealed a 3-factor solution with an explained variance of 60.52% confirmed by parallel analysis. With CFA, fit indices were found to be acceptable for first and second-order models (X²/df=4,0741; CFI =.8638; TLI=.8382; GFI=.8515; RMSEA= .0887, p<.001). Cronbach’s alphas were of .871 for the total scale (16 items), .857 for F1 (Collective actions/CA; 5 items), .832 for F2 (Personal behaviours/PB; 8 items) and .736 for F3 (Political actions/PA; 3 items). PEBS correlated with CC-DIS (r=.48 with total PEBS, r=.42 with CA and PB and r=.21 with PA, p<.01), ProBeQ (r=.24 with total PEBS, r=.12 with CA, r=.37 with PB and r=-.14 with PA, p<.01), Narcissistic Perfectionism (r=.08, p<.05 with CA and r=.20, p<.01 with PA), Honesty-Humility (r=-.15, p<.01 with PA), Emotionality (r=-.12, p<.01 with PB and r=.09, p<.05 with PA), Extraversion (r=.09, p<.05 with CA and r=.12, p<.01 with PA), Agreeableness (r=.11 with total PEBS, r=.12 with CA and r=.14 with PA, p<.01) and Conscientiousness (r=.09, p<.05 with total PEBS). When the correlated variables were inserted as predictors in linear multiple regression models where CA, PB and PA were dependent variables, they explained 19.8% (R^2^=.198), 24.6% (R^2^=.246) and 14.9% (R^2^=.149) of their variance, respectively (all p<.001).

**Conclusions:**

PEBS shows adequate psychometric properties, therefore, it can be used to measure PEB in the Portuguese population, namely, to analyze the efficacy of pro-climate interventions and campaigns. These initiatives should take into consideration the role of individual factors (such as personality traits) in PEB.

**Disclosure of Interest:**

None Declared

